# Eudaimonic life values and employment stress: a moderated mediation study in Chinese higher education students

**DOI:** 10.3389/fpsyg.2026.1768637

**Published:** 2026-04-10

**Authors:** Xiao Ke, Qianhan Huang, Qi Chen, Xi Pei

**Affiliations:** 1 Shenzhen University, Shenzhen, China; 2 Shenzhen Polytechnic University, Shenzhen, China

**Keywords:** employment mindset, employment stress, eudaimonic life values, higher education students, moderated mediation

## Abstract

**Introduction:**

This study examined how eudaimonic life values (ELV) influence employment stress (ES) among Chinese higher education students, focusing on the mediating role of employment mindset (EM) and the moderating effects of subject major and educational background.

**Methods:**

A cross-sectional survey was conducted from February to April 2025 among students aged 18–25 from 18 higher education institutions in Shenzhen, China. A total of 9,264 valid responses were analyzed using mediation and moderated mediation models.

**Results:**

ELV significantly and negatively predicted both EM (*β* = −0.073, *p* < .001) and ES (*β* = −0.009, *p* < .001), while EM positively predicted ES (*β* = 0.125, *p* < .001). The indirect effect of ELV on ES via EM was significant (95% CI [−0.0102, −0.0082]), indicating partial mediation, with 50.8% of the total effect mediated. Both direct and indirect effects were moderated by subject major and educational background, with stronger effects observed among natural sciences and engineering students and those with higher educational levels.

**Discussion:**

Findings highlight the role of cognitive-emotional processes in linking life values to employment stress and suggest that educational context shapes these relationships.

## 1 Introduction

### 1.1 Background

The mass expansion of higher education has significantly complicated the employment landscape for graduates in recent years (Figueiredo et al., 2015). This situation has been further exacerbated by the COVID-19 pandemic, which intensified structural mismatches and introduced new challenges, including a sharp contraction in recruitment demand and fiercer competition for fewer positions (Wang and Wang, 2024). Consequently, graduates face a considerable psychological burden. These challenges are not confined to the global level but are also evident in the Chinese context. In recent years, the employment difficulties faced by Chinese college graduates have not only reflected local economic conditions but also embodied broader issues such as global value chain contraction and credential inflation within the Chinese labor market (Knight et al., 2017; Pinna and Lodi, 2021). A growing body of research suggests that employment mindset—graduates’ beliefs and attitudes toward the employment process—often reflects varying degrees of employment-related anxiety and has emerged as a key contributor to perceived stress (Belle et al., 2022; Wan et al., 2023).

In light of these challenges, psychological resources are increasingly recognized as crucial in mitigating perceived stress. However, existing studies have largely focused on surface-level protective factors such as life satisfaction and resilience (Pasupuleti et al., 2009; Wang and Zhang, 2025). While these constructs are important, they may not fully capture the deeper psychological mechanisms that shape how individuals experience employment-related stress. Building upon this gap, the present study highlights the role of eudaimonic life values—defined as a fundamental orientation grounded in satisfaction with the present, optimism about the future, and commitment to ideals and social values (Busseri, 2022). In Western psychology, eudaimonia is typically conceptualized as the pursuit of meaning, personal growth, and self-realization (Ryan et al., 2008). However, the meaning of well-being and life values may vary across cultural contexts. Research has shown that in Chinese culture, individuals’ understanding of happiness and meaningful life often extends beyond personal fulfillment to include relational harmony, social responsibility, and identification with collective development (Brailovskaia et al., 2021; Steele and Lynch, 2012). Studies on Chinese students further suggest that well-being may involve multiple levels—including individual, relational, and societal dimensions—reflecting the influence of both traditional collectivist values and modern aspirations for personal development (Xu et al., 2024). Therefore, in the present study, eudaimonic life values are conceptualized as a value orientation integrating personal ideal pursuit, social contribution, and confidence in broader societal development. Extending Zhao et al.’s (2023) findings, which identified domain-specific mindsets (e.g., intelligence or stress mindsets) as proximal predictors of perceived stress, this study proposes that eudaimonic values serve as a more stable and foundational source of these mindsets. Specifically, it is posited that eudaimonic life values negatively predict employment stress, both directly and indirectly through their influence on employment mindset. This mediating mechanism suggests that values shape how individuals interpret and cope with employment-related challenges, offering a more integrative framework for understanding individual differences in stress perception.

In addition to general psychological resources, employment stress may also vary according to individual characteristics. Prior research suggests that both educational background (e.g., college, undergraduate, postgraduate) and academic discipline (e.g., humanities and social science vs. natural sciences and engineering) are associated with distinct employment experiences and coping resources (Breetzke et al., 2023; Karaca-Atik et al., 2023). These contextual dimensions not only influence graduates’ perceptions of employment stress but may also shape how their underlying values translate into specific attitudes and coping styles. Therefore, this study further explores whether educational background and subject moderate the association between the eudaimonic life values and employment mindset, as well as the direct link between the perception of a better life and employment stress. In other words, the indirect and direct effects of value orientation on employment stress may vary across different educational and disciplinary contexts.

### 1.2 Eudaimonic life values and employment stress

In Western psychological research, the eudaimonic life values can be conceptualized as an orientation that highlights the pursuit of intrinsically valued goals such as personal growth, deep relationships, community contribution, and health (Ryan et al., 2008). In the Chinese context, however, this orientation carries distinct cultural characteristics, including the pursuit of ideals, confidence in future development through individual effort, and a commitment to self-realization by contributing to society (Jin and Wang, 2025). Unlike well-established constructs such as subjective well-being, eudaimonic life values has not yet been accompanied by a standardized scale. To address this gap, the present study employs a measure modified from (Gao et al., 2020) to ensure cultural relevance, operationalizing the construct in terms of satisfaction with current life conditions, confidence in the future, and adherence to ideals and social value. In contrast, employment stress is typically defined as the negative emotional and cognitive experiences that arise during the job-seeking process (da Motta Veiga and Turban, 2014). From this perspective, individuals who endorse a stronger orientation toward a better life are less likely to tie their self-worth to immediate job outcomes or external evaluations. Instead, they focus on inner value realization and broader life goals, which enables them to view setbacks in the employment process with greater perspective (Cheng, 2024). This orientation reduces their susceptibility to employment stress by shifting attention away from short-term frustrations and toward long-term growth.

Although few studies have directly examined the relationship between the eudaimonic life values and employment stress, existing evidence suggests that positive psychological resources can buffer job-seeking pressure. For example, da Motta Veiga and Turban (2014) found that job seekers with a “Learning Goal Orientation” as a value-based resource were able to transform employment stress into stronger job-search motivation rather than being overwhelmed by it. In addition, Lim et al. (2016) found that unemployed individuals with value-oriented psychological resources such as hope and optimism reported lower levels of employment stress (or fatigue) and were more likely to secure high-quality jobs. These findings indirectly suggest that a better life orientation can function as an important psychological resource to help individuals cope effectively with stress. Therefore, it is reasonable to hypothesize that a stronger sense of the eudaimonic life values will be directly associated with lower perceived employment stress.

### 1.3 The mediating role of employment mindset

According to Sandler’s (2001) self-system belief theory, self-system beliefs are key personal resources that shape individuals’ fundamental self-perceptions and evaluations in interaction with their environment. These beliefs mediate the impact of external contexts on psychological adjustment, particularly in terms of self-worth, sense of control, and social belonging. In the employment context, individuals develop job-related self-system beliefs (i.e., employment mindset), which guide their evaluation of whether the environment meets their basic psychological needs for competence, autonomy, and relatedness. When these needs are fulfilled, a positive employment mindset is more likely to emerge, thereby buffering the negative effects of employment stress. Recent studies in China have increasingly emphasized the importance of employment mindset in understanding university students’ employment-related psychological adjustment. Empirical research suggests that employment mindset reflects individuals’ cognitive evaluations and coping orientations toward labor market uncertainty and career development, which can significantly influence perceived employment stress and career decision-making (Shen et al., 2025; Xu et al., 2022).

Thus, it can be inferred that employment mindset, as an expression of self-system beliefs, may mediate the relationship between a flourishing life orientation (as a positive psychological resource) and employment stress (Keating and Heslin, 2015), such that a flourishing life orientation promotes a positive employment mindset, which in turn reduces perceived stress. Although direct tests of the “eudaimonic life values” → employment mindset → employment stress” pathway remain scarce, multiple lines of functionally equivalent evidence consistently demonstrate that positive psychological resources influence employment anxiety/stress through self-system beliefs (Shen et al., 2025). This provides transferable empirical support for the hypothesis that the eudaimonic life values life may alleviate employment stress via employment mindset.

A growing body of empirical studies has provided evidence for this proposed mediating mechanism. For example, Yang and Su (2025) has found that that positive psychological resources can strengthen individuals’ self-system beliefs (such as employment mindset), thereby facilitate positive career adaptation outcomes and potentially reduce employment stress. In addition, empirical research demonstrated that psychological capital, as a positive psychological resource, significantly reduced employment anxiety by enhancing self-management (i.e., the executive facet of employment mindset). Notably, the mediating effect of self-management accounted for 30.22% of the total effect (Shi et al., 2023). Survey evidence among higher education students found that social support can both directly alleviate employment anxiety and indirectly reduce it by enhancing self-efficacy (Gao et al., 2020). Since self-efficacy is a core indicator of self-system beliefs (Sandler, 2001), this finding further confirms that when external positive resources (such as social support or the eudaimonic life values) strengthen individuals’ self-system beliefs, employment stress decreases accordingly. In conclusion, based on self-system belief theory and supporting empirical evidence, it is reasonable to hypothesize that employment mindset mediates the relationship between the perception of a better life and employment stress.

### 1.4 The moderator of educational background and subject

According to Hobfoll’s Conservation of Resources (COR) theory, stress arises from the threat or loss of valuable resources, and the amount and type of resources individuals possess affect their coping ability (Hobfoll, 1989). Within this framework, education level (vocational college, undergraduate, postgraduate) and disciplinary background (humanities and social sciences vs. natural sciences and engineering) can serve as key moderators. Higher education levels can provide individuals with richer knowledge, skills, social capital, and economic resources—assets that may enhance their capacity to manage challenges and serve as potential tools for coping with stress. Empirical studies provide support for this perspective. For instance, research indicates that education level is an important moderator of work-related stress. Specifically, when facing highly repetitive tasks, individuals with higher education can better mobilize cognitive resources to treat such tasks as a form of variation, resulting in lower stress. Conversely, when confronted with unemployment threats or stalled career advancement, highly educated individuals perceive a greater risk of resource loss, and their stress levels may be higher than those of less-educated peers (Schoger, 2023). In China, existing research has indicated that in the context of rapid transformation driven by emerging technologies, educational attainment not only determines graduates’ initial employment opportunities but also alleviates their job stress through dual pathways of skill enhancement and labor market signaling (Chen, 2025). Specifically, postgraduate education, compared with undergraduate degrees, better equips graduates for professional and technical positions and higher income levels, thereby buffering employment anxiety caused by external shocks.

Additionally, disciplinary background shapes resource specificity: natural sciences and engineering develop analytical and technical skills suited to objective challenges, while humanities cultivate social and reflective skills that help navigate interpersonal or value-related stress (Karaca-Atik et al., 2023). COR theory emphasizes that resources are most effective when matched to the stressor, suggesting that education and discipline may moderate individuals’ vulnerability or resilience to employment stress. Over time, different disciplines foster distinct cognitive frames, which shape how graduates interpret a good life and employment setbacks. Consequently, even under the same level of positive expectations, these cognitive frames lead to heterogeneous stress appraisals. From a broader social perspective, disciplinary effects are further shaped by contextual factors. In technology-oriented big cities such as Shenzhen, China, where the labor market and social values prioritize innovation and technical expertise (Cheng et al., 2013), graduates from science and engineering fields tend to have skills that align more closely with dominant employment demands. In contrast, humanities graduates—whose strengths lie in reflection and communication—may experience a resource–environment mismatch, leading to greater perceived stress despite similarly positive life values. Thus, the moderating role of discipline reflects both psychological resource specificity and a socio-economic context that favors certain forms of educational background.

In this sense, education level and disciplinary background can be viewed as contextual resources that not only condition how individuals translate positive life beliefs into employment mindset (X → M), but also how such beliefs directly influence employment stress (X → Y). However, once internal self-system beliefs are established, their impact on stress (M → Y) primarily reflects intra-individual processes, which are less contingent on educational or disciplinary differences. Therefore, we focus on moderation in the first stage and direct path, but not in the second stage.

### 1.5 The present study

The traditional practice of treating graduates as a homogeneous group fails to capture key differences in their career trajectories, particularly in terms of employment stress. Existing studies rarely take into account that educational background (e.g., college, undergraduate, postgraduate) and disciplinary field (e.g., humanities and social science vs. natural sciences and engineering) may shape individuals’ perception of life and employment experiences in distinct ways (Breetzke et al., 2023; Pan et al., 2025). At the same time, prior research has largely overlooked the deeper psychological mechanisms through which values affect employment stress, often limiting the analysis to surface-level variables such as life satisfaction and resilience. To address these gaps, the present study draws on Conservation of Resources (COR) theory (Hobfoll, 1989) and the self-system belief framework (Sandler, 2001) to argue that the eudaimonic life values, as fundamental psychological resources, may play a critical role in shaping stress perception. These perspectives further suggest that such effects are unlikely to be uniform but may vary depending on contextual factors such as educational background and subject. Therefore, it is imperative to move beyond a one-size-fits-all analytical framework and explicitly examine both the underlying psychological mechanisms and the moderating roles of educational background and subject, the present study hypothesizes that:

*H1*: The eudaimonic life values directly and negatively predict employment stress.

*H2*: Employment mindset mediates the relationship between the eudaimonic life values and employment stress.

*H3*: The negative relationship between eudaimonic life values and employment stress will vary across educational backgrounds and disciplinary fields.

*H4*: The mediating effect of employment mindset between eudaimonic life values and employment stress will differ across educational backgrounds and disciplinary fields.

## 2 Method

### 2.1 Participants and procedure

This large-scale survey targeted higher education students aged 18–25 across 18 higher education institutions in Shenzhen, China—a major innovation-driven, first-tier city. Participants were drawn from multiple educational levels within the Chinese higher education system, including vocational college (junior college), undergraduate, and graduate programs. The study was designed to capture a broad and diverse cross-section of the student population in a rapidly developing urban context. The participating institutions included colleges, comprehensive universities. Data were collected from February to the end of April 2025. The survey was administered online through Wenjuanxing, a widely used Chinese survey platform. Participation was voluntary and anonymous, and informed consent was obtained from all participants before data collection. To ensure data quality, multiple screening criteria were applied. First, instructed response items were embedded (e.g., requiring participants to choose a specified option); respondents who failed these items were excluded. Second, participants who selected the same response option for more than half of the items consecutively were removed to avoid patterned responding. Third, questionnaires completed in less than twice the number of items in seconds (i.e., fewer than 2-s per item) were considered invalid and excluded from the final dataset. Ethical approval for this study was granted by the Medical Ethics Committee of Shenzhen Polytechnic University (Approval No. 20250925-09), and the study was conducted in accordance with the Declaration of Helsinki.

A total of 10,569 questionnaires were collected, of which 9,264 were deemed valid, resulting in an effective response rate of 87.6%. Participants came from diverse majors and grade levels, ensuring representativeness across different academic backgrounds.

### 2.2 Measure

#### 2.2.1 Eudaimonic life values

This variable was measured using a self-constructed scale adapted from University Students’ Life Value Questionnaire developed by Gao (2023). Drawing on the original framework, six items were selected and modified to capture key dimensions such as idealistic orientation, social value identification, life satisfaction, and confidence in achieving personal goals. Representative items include: “I believe life should have ideals and the continuous pursuit of long-term goals,” and “I am confident that I can ultimately achieve a better life through my own efforts.” Participants rated each item on a 5-point Likert scale (1 = strongly disagree, 5 = strongly agree). The adapted scale demonstrated good internal consistency (Cronbach’s *α* = 0.83). Confirmatory factor analysis (CFA) supported the scale’s unidimensional structure, yielding satisfactory model fit indices: CFI = 0.992, TLI = 0.983, SRMR = 0.013.

#### 2.2.2 Employment mindset

This variable was measured using a single self-developed item: “What is your current employment mindset?” Responses were recorded on a 4-point Likert scale ranging from 1 (“very confident in finding a job”) to 4 (“very confused about employment”). A single-item indicator was adopted to capture participants’ overall evaluation of their current employment mindset and to reduce respondent burden in this large-scale survey. Previous research suggests that single-item measures can be acceptable when assessing relatively concrete and global perceptions in large population-based studies (Wanous et al., 1997; Bergkvist and Rossiter, 2007). Higher scores reflected greater levels of employment-related confusion. In this study, the mean score was 2.71 (SD = 1.15).

#### 2.2.3 Employment stress

Employment stress was assessed with a single item: “How much employment stress do you feel you are facing?” Participants responded on a 3-point Likert scale ranging from 1 (“no stress”) to 3 (“high stress”). Higher scores indicated greater perceived employment-related stress. Consistent with previous large-scale survey research, a single-item measure was used to capture participants’ overall perceived employment stress while minimizing questionnaire length and respondent burden (Wanous et al., 1997; Bergkvist and Rossiter, 2007). The mean score in this study was 2.54 (SD = 0.54).

### 2.3 Statistical analysis

Descriptive analyses were first conducted to summarize the sociodemographic characteristics of the participants. Correlation analyses were then performed to examine the associations among the study variables. Mediation and moderated mediation analyses were carried out using the PROCESS macro v4.2. for SPSS 25 (Hayes, 2022), with 5,000 bootstrapped resamples to generate bias-corrected 95% confidence intervals (CIs).

To begin with, Model 4 was employed to test whether employment mindset mediated the relationship between students’ eudaimonic life values and employment stress. A mediating effect was considered significant if the 95% CI of the indirect effect (path a*b) did not include zero. Next, Model 8 was applied to explore the moderated mediation model, in which gender was specified as a moderator of the indirect pathway. A significant moderated mediation effect was established if the 95% CI for the interaction term excluded zero.

All analyses were conducted using SPSS version 26.0, and gender was included as the only covariate in the models.

## 3 Result

### 3.1 Descriptive statistics

Descriptive statistics for the primary study variables are summarized in Table 1. A total of 9,264 participants were included in the final analysis. Of these, 5,403 were male (58.3%) and 3,861 were female (41.7%). With respect to academic discipline, 43.9% of the respondents majored in humanities and social sciences (e.g., economics, law), whereas 56.1% specialized in natural sciences and engineering (e.g., agriculture, engineering). Regarding educational background, the proportions of vocational college students and undergraduates were nearly identical, each comprising 44.4% of the sample. An additional 11.3% were either currently enrolled in or had completed graduate-level programs, including master’ s and doctoral degrees.

**Table 1:** Sociodemographic characteristics.

Characteristics	Total (*n* = 9,264)			
	*N*	%	Mean	Std.
Gender				
Female	3,861	41.7		
Male	5,403	58.3		
Subject				
Humanities and social science	4,068	43.9		
Natural sciences and engineering	5,196	56.1		
Educational background				
Vocational college	4,109	44.4		
Undergraduate	4,111	44.4		
Graduate	1,044	11.3		
ELV scores			24.60	3.84
ES scores			2.54	0.54
EM scores			2.71	1.15

ELV, eudaimonic life values; ES, employment stress; EM, employment mindset.

### 3.2 Correlation analysis

Means, standard deviations, and Pearson correlation coefficients for the main study variables are reported in Table 2. As shown, the total score of the eudaimonic life values (ELV) was significantly correlated with both Employment Mindset (EM) and Employment Stress (ES), suggesting that youth values are meaningfully associated with career-related attitudes and perceived stress.

**Table 2:** Correlation matrix for key variables.

Variable	1	2	3
1. ELV	1		
2. ES	−0.126**	1	
3. EM	−0.243**	0.282**	1

ELV, eudaimonic life values; ES, employment stress; EM, employment mindset.

**p < 0.01.

### 3.3 Mediation analysis

The mediation analysis (see Table 3) showed that ELV significantly predicted EM (*β* = −0.073, *p* < 0.001), which in turn positively predicted ES (*β* = 0.125, *p* < 0.001). The direct effect of ELV on ES remained significant (*β* = −0.009, *p* < 0.001), while the indirect effect via EM was also significant (effect = −0.009, 95% CI [−0.0102, −0.0082]). These results suggest that the relationship between ELV and ES was partially mediated by EM. Further analysis indicated that EM played a substantial mediating role: 50.8% of the total negative effect of ELV on ES was transmitted through EM, underscoring its importance in linking life perception to employment stress.

**Table 3:** Mediation analysis.

Path	*β*	*SE*	*t*	*p*	Effect	95% CI (LL, UL)
ELV → EM	−0.073	0.003	−24.07	<0.001		[−0.079, −0.067]
EM → ES	0.125	0.005	26.05	<0.001		[0.116, 0.135]
ELV → ES (Direct Effect)	−0.009	0.001	−5.99	<0.001		[−0.012, −0.006]
ELV → ES (Total Effect)	−0.018	0.001	−12.25	<0.001		[−0.021, −0.015]
ELV → ES (Indirect Effect)					−0.009	[−0.010, −0.008]

In all statistical analyses, gender was included as a control variable.

### 3.4 Moderated mediation analysis

**Figure 1:**
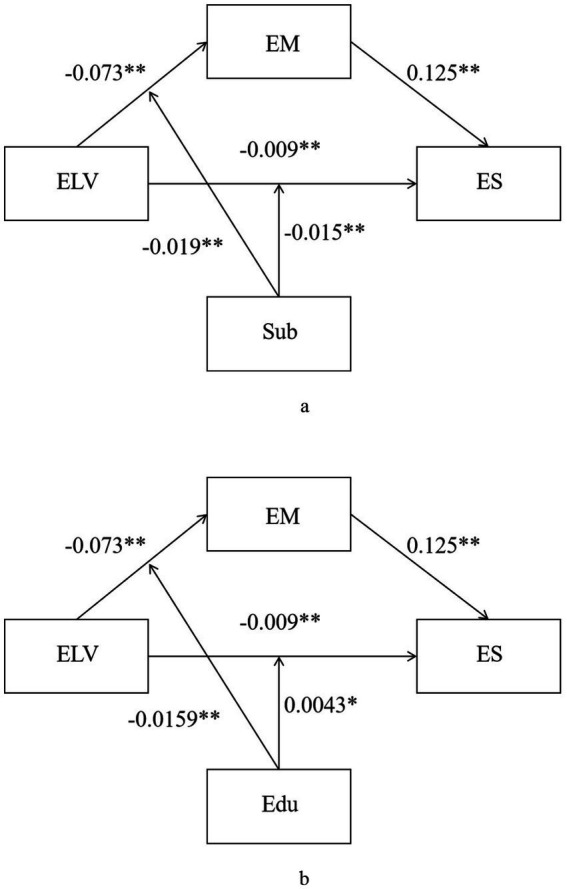
Path check result graph. ELV, eudaimonic life values; EM, employment mindset; ES, employment stress; sub, subject; Edu, educational background. **p < 0.01; *p < 0.05. **(a)** presents the path coefficients when subject serves as the moderator variable, and **(b)** presents the path coefficients when educational background serves as the moderator variable.

**Figure 2:**
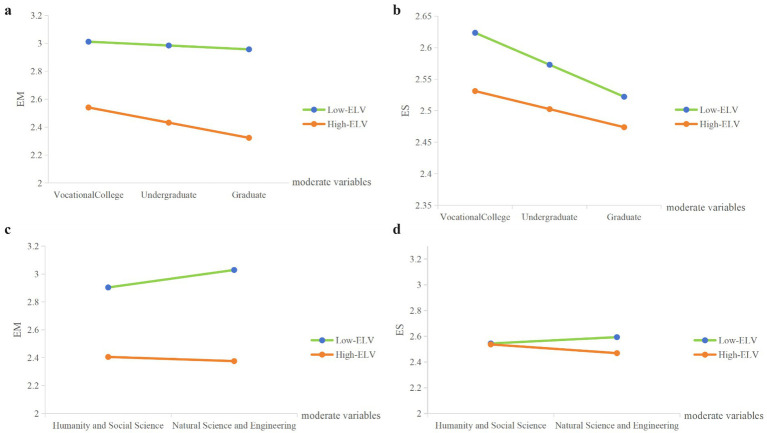
Results of simple slope test. **(a,b)** present the simple slope analyses of employment mindset (EM) and employment stress (ES), respectively, with subject as the moderator. **(c,d)** present the simple slope analyses of EM and ES, respectively, with educational background as the moderator. Low-ELV refers to scores one standard deviation below the mean, and High-ELV refers to scores one standard deviation above the mean.

A moderated mediation analysis was conducted with life values as the predictor, employment mindset as the mediator, and employment stress as the outcome, controlling for gender (see Table 4). In Model 1 (see Figure 1a), where subject major was tested as the moderator, significant interaction effects were observed on both the path from life values to employment mindset (*β* = −0.019, SE = 0.006, *t* = −3.23, *p* = 0.001) and the path from life values to employment stress (*β* = −0.015, SE = 0.003, *t* = −5.21, *p* < 0.001). Simple slope tests further revealed that the negative effect of life values on employment mindset was stronger among natural sciences and engineering students (*β* = −0.083, *p* < 0.001) compared with humanities and social sciences students (*β* = −0.062, *p* < 0.001). Moreover, the direct negative effect of life values on employment stress was significant only for natural sciences and engineering students (*β* = −0.016, *p* < 0.001) but not for humanities/social sciences students (*β* = −0.001, *p* = 0.663). The simple slope plots are shown in Figures 2a,b.

**Table 4:** Moderation analysis.

Path	*b*	*SE*	*t*	*p*	95% CI [LL, UL]
Model 1					
ELV × Subject → EM	−0.019	0.006	−3.23	0.001	[−0.031, −0.008]
ELV × Subject → ES	−0.015	0.003	−5.21	< 0.001	[−0.020, −0.009]
Conditional indirect effects (Bootstrapped)					
Humanities and social sciences	−0.008	0.001	–	–	[−0.009, −0.007]
Natural sciences and engineering	−0.010	0.001	–	–	[−0.011, −0.009]
Index of moderated mediation	−0.0024	0.0008	–	–	[−0.0039, −0.0008]
Model 2					
ELV × Edu → EM	−0.0159	0.0042	−3.78	< 0.001	[−0.0241, −0.0076]
ELV × Edu → ES	0.0043	0.0020	2.19	0.028	[0.001, 0.008]
Conditional indirect effects (Bootstrapped)					
Vocational College	−0.0075	0.0006			[−0.0088, −0.0063]
Undergraduate	−0.0089	0.0005			[−0.0099, −0.0079]
Graduate	−0.0102	0.0006			[−0.0114, −0.0090]
Index of moderated mediation	−0.0020	0.0005			[−0.0030, −0.0009]

In all statistical analyses, gender was included as a control variable.

In Model 2 (see Figure 1b), where educational background was tested as the moderator, significant interactions also emerged on both the mediator path (*β* = −0.016, *SE* = 0.004, *t* = −3.78, *p* < 0.001) and the direct path (*β* = 0.004, *SE* = 0.002, *t* = 2.19, *p* = 0.028). Simple slope analyses showed that the negative association between life values and employment mindset became progressively stronger with higher levels of education (college: *β* = −0.061; undergraduate: *β* = −0.072; graduate: *β* = −0.083; all ps < 0.001). Conversely, the negative relationship between life values and employment stress weakened as education level increased (college: *β* = −0.012, *p* < 0.001; undergraduate: *β* = −0.009, *p* < 0.001; graduate: *β* = −0.006, *p* = 0.001). For both models, the indices of moderated mediation were significant (subject major: Index = −0.0024, 95% CI [−0.0039, −0.0008]; educational background: Index = −0.0020, 95% CI [−0.0030, −0.0009]). These results indicate that the indirect effect of life values on employment stress via employment mindset differed significantly across subject majors and educational levels. The simple slope plots are shown in Figures 2c,d.

## 4 Discussion

This study constructed a moderated mediation model to examine the underlying mechanism through which the eudaimonic life values (ELV) influences employment stress (ES). Specifically, it investigated the mediating role of employment mindset (EM) and the moderating effects of subject and educational background. The findings supported the proposed hypotheses: employment mindset partially mediated the relationship between eudaimonic life values and employment stress, while both subject and educational background moderated the first stage of the mediation and the direct path from ELV to ES.

The results show that ELV is a significantly negative predictor of employment stress. The significant direct effect of ELV on employment stress highlights the protective role of positive life values. Although the magnitude of this direct effect is relatively small, such effect sizes are not uncommon in large-scale psychological survey research. Even modest effects may carry practical significance when considering the large number of graduates facing employment-related stress during the school-to-work transition. This finding aligns with previous research suggesting that eudaimonic life values provide a stable cognitive and motivational foundation that fosters psychological resilience and emotional balance under stress (Busseri, 2022; Joshanloo, 2019). Individuals who pursue intrinsically meaningful goals—such as personal growth, contribution, and self-realization—tend to maintain a stronger sense of purpose and control when facing uncertainty. Similarly, studies on positive psychological orientations show that traits like optimism, hope, and gratitude are linked to lower stress and better emotional well-being (Romswinkel et al., 2018; Aldbyani et al., 2025). Together, these findings suggest that eudaimonic life values act as enduring psychological resources that help individuals regulate stress and sustain well-being. In China’s highly competitive job market, such value systems—integrating personal ideals with social commitment—may play a crucial role in mitigating employment-related stress.

The mediating effect of employment mindset aligns with the self-system belief framework (Sandler, 2001), which posits that individuals’ core beliefs shape their interpretations of and reactions to stressful situations. In this study, eudaimonic life values were found to influence employment mindset—a more proximal belief system—further indirectly affecting stress perceptions. Graduates with strong eudaimonic life values are more likely to develop growth-oriented employment mindsets (Kondratowicz and Godlewska-Werner, 2022), characterized by confidence in self-improvement, openness to learning, and adaptability during the job search process. This finding is consistent with theoretical perspectives suggesting that individuals’ mindsets shape how they interpret and respond to stress. A constructive or growth-oriented mindset promotes proactive coping and positive reappraisal, thereby reducing perceived stress and enhancing well-being (Keech et al., 2018). In this sense, employment mindset serves as a key self-system belief that translates deep-seated values into adaptive stress responses, clarifying the internal mechanism through which eudaimonic life values influence employment stress.

Furthermore, the moderated mediation analysis revealed that both subject and educational background shaped the strength of the indirect and direct effects of ELV on ES. Specifically, the negative associations between ELV and EM, as well as between ELV and ES, were stronger among students in the natural sciences than those in the humanities and social sciences. Moreover, the indirect effect through EM increased with higher levels of education, whereas the direct effect of ELV on ES weakened. These patterns suggest that disciplinary and educational contexts influence how individuals interpret and respond to stress in relation to their life values. Higher educational attainment may enhance individuals’ coping resources and cognitive flexibility, thereby reducing the direct impact of value orientations on stress experiences while amplifying the role of mediating factors such as meaning or mindset (Agai-Demjaha et al., 2015; Schoger, 2023; Qin et al., 2023). Similarly, students in the natural sciences and engineering may face more performance-oriented or competitive learning environments, which could intensify the stress effects of their value orientations and emotional mindsets.

These findings can be interpreted through the lens of Conservation of Resources (COR) theory (Hobfoll, 1989), which posits that stress arises when individuals perceive a loss or threat to valued resources. The theory also emphasizes that the type and relevance of available resources determine how effectively individuals can cope with specific stressors. In summary, these moderated mediation results suggest that the relationship between values, mindset, and stress is contextually embedded. Both subject and education influence how effectively individuals mobilize psychological resources derived from their value systems. Integrating COR theory and the self-system belief framework thus provides a comprehensive explanation of why the same life values may yield different stress outcomes across educational and disciplinary contexts. More specifically, these differences may reflect variations in disciplinary training and resource expectations. Science and engineering programs often emphasize problem-solving and technical skills that align closely with labor market demands, which may help students translate positive life values into adaptive employment mindsets. By contrast, humanities and social science programs tend to emphasize reflective and communicative competencies that may be less directly linked to technology-driven job markets (Kuo and Roscigno, 2025; Qu et al., 2024). In addition, higher educational attainment is often associated with greater knowledge resources and professional opportunities, but also with higher career expectations (Georgios et al., 2025). When employment outcomes fall short of these expectations, highly educated students may experience stronger stress responses.

## 5 Limitations and future directions

Several limitations should be noted. First, although the moderated mediation model specifies directional paths based on theory, the cross-sectional nature of the data limits causal inference. Therefore, the relationships identified in this study should be interpreted as statistical associations rather than definitive causal effects. Future research could employ longitudinal or experimental designs to examine how these variables dynamically influence one another over time. In addition, although gender was controlled for in the analysis, other potential confounding variables—such as grade level, internship experience, and family socioeconomic status—were not included. Future research could incorporate a broader range of contextual variables to provide a more comprehensive understanding of factors influencing employment stress. Second, as empirical research on the eudaimonic life values remains limited, the operationalization of this construct in the current study may not fully capture its conceptual breadth and depth. Future studies should refine measurement tools and explore the multidimensional nature of life values across different cultural and developmental contexts. Third, all data were obtained through self-report questionnaires, which may involve subjective interpretation and response bias. Future research could enhance measurement validity by incorporating multi-source or behavioral data, such as peer evaluations or physiological indicators of stress. Moreover, employment mindset and employment stress were measured using single-item indicators, which may limit the ability to fully capture the complexity of these constructs. Future studies could employ multi-item validated scales to improve measurement reliability and provide a more comprehensive assessment. Fourth, although the moderated mediation analysis revealed statistically significant moderating effects of subject major and educational background, these findings should be interpreted with caution. Given the large sample size, even small effect sizes may reach significance due to increased statistical power. While the confidence intervals were consistent and the patterns reliable, the practical significance of these effects remains limited. This highlights the need to avoid overinterpretation of minor moderating effects and instead consider them as preliminary indicators of potential contextual influences. Future research may further explore these patterns by incorporating additional social and psychological moderators, ideally across different populations and methodological designs. Despite these limitations, the present findings contribute novel insights into the psychological mechanisms linking life values and employment stress, offering a theoretical and empirical foundation for future research on value-based stress regulation among young adults.

## 6 Conclusion

This study constructed a moderated mediation model to examine how life values influence employment stress among graduates. The findings revealed that the eudaimonic life values negatively predicted employment stress, both directly and indirectly through employment mindset. Moreover, subject and educational background moderated the strength of these relationships, indicating that the effects of eudaimonic life values on stress are shaped by individuals’ disciplinary and educational contexts. These findings highlight the importance of value-based psychological resources in stress adaptation during the school-to-work transition. From a practical perspective, universities should incorporate value-oriented career education into career development programs to help students clarify life goals and strengthen meaning-oriented life values. In addition, career guidance services may benefit from interventions that cultivate positive employment mindsets, such as resilience training and adaptive coping strategies for job search challenges. Considering the moderating effects identified in this study, universities may also provide more differentiated career guidance for students from different disciplinary and educational backgrounds. Such efforts may help graduates better translate personal psychological resources into effective coping strategies when facing employment stress.

## Data Availability

The datasets generated and analyzed during the current study are not publicly available due to privacy and ethical restrictions. Requests to access the datasets should be directed to peixi@szpu.edu.cn.
